# Phytochemical Analysis and Metal-chelation Activity of *Achillea tenuifolia* Lam.

**Published:** 2012

**Authors:** Shirin Moradkhani, Abdul Majid Ayatollahi, Mustafa Ghanadian, Mohammad Reza Moin, Masoud Razavizadeh, Mohsen Shahlaei

**Affiliations:** a *School of Pharmacy, Shaheed Beheshti University of Medical Sciences, Tehran, Iran. *; b *Phytochemistry Research Center, Shahid Beheshti University of Medical Sciences, Tehran, Iran.*; c *Pharmaceutical Sciences Research Center, Isfahan University of Medical Sciences, Isfahan, Iran.*; d *Faculty of Pharmacy, Shiraz University of Medical Sciences, Shiraz, Iran.*; e *Ministry of Health and Medical Education, Iran.*; f *Department of Medicinal Chemistry, Faculty of Pharmacy, Kermanshah University of Medical Sciences, Kermanshah, Iran.*; g *Student Research committee, School of Pharmacy, Shahid Beheshti University of Medical Sciences, Tehran, Iran. *

**Keywords:** *Achillea tenuifolia*, Iron overload, 5-hydroxy-flavone, Quercetin, Chelation assay

## Abstract

*Achillea tenuifolia *Lam. (Asteraceae) afforded a dichloromethane fraction from which three known compounds *β*-sitosterol (compound1), 5-hydroxy, 4*'*,6,7– trimethoxy flavone (salvigenin compound 2), and methyl-gallate (compound 3) were isolated for the first time. The structure of isolated compounds was elucidated by different spectroscopic methods. Applying the molar-ratio method, the complexation of salvigenin with Fe (III), Cu(II) and Zn(II), the most abundant type of metal ions in the body, were then evaluated. It was determined that stoichiometric ratio of salvigenin with these cations were as Fe(Salvigenin)_2_ (H2O)_2_ and Cu(Salvigenin)_2_(H2O)_2_ in methanolic solution without pH control, while zinc ions didn`t form significant complexes. The results were confirmed more, by computational molecular modeling of the structure of proposed ligand-complexes by semi-imperical PM3 calculations, which determined negative heat of formation for the complexes Fe(III) and Cu(II) ions as -689.7 and -573.5, respectively and proposed chelating affinity of salvigenin in the following order: Fe(III) > Cu(II) >> Zn(II).

## Introduction

Asteraceae, the largest family of angiosperms, comprises about 1500 genera and 23000 species, distributed in three subfamilies and seventeen tribes. The genus *Achillea *is composed of 115 species of perennial herbs, all native to temperate regions of the northern hemisphere (1). Aerial parts of different species of this genus are widely used in folk medicine for preparation of herbal teas with antiphlogistic and spasmolytic activity (2). One document published about two centuries ago as Makhzan-ol-Advieh, recommended it for bladder stone and urinary obstruction (3). Recent studies show that plant extracts exhibit pharmacological activities like anti-inflammatory and antiallergic (2), antihelmintic, cholagogue, antibacterial and antioxidant properties (4). *Achillea tenuifolia *Lam. is a perennial herb, distributed in some regions of Iran (5). In previous studies, the preliminary chemical examination has demonstrated that phenolics compounds were responsible for the antioxidant and lipid peroxidation inhibitory effects of this plant (6-8). Therefore, considering the recently pharmacological studies and introduction of some flavonoids in iron-chelating therapy for diseases like thalassemia (9), the *in-vitro *iron-chelating effect of flavonoid isolated from *Achillea tenuifolia *was studied to provide further support for flavonoids to be candidate for iron overload disorders.

**Figure 1:**
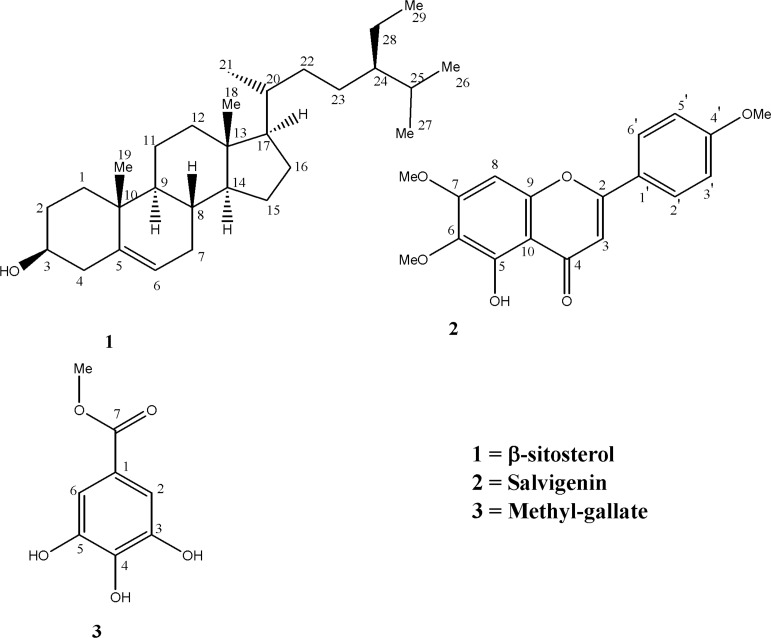
Structures of the isolated compounds (13-) from *Achillea tenuifolia*

## Experimental

*General*

The NMR spectra were recorded on a Bruker Avance AV 400 and AV 500 NMR instrument. Infrared spectra were recorded on a *FTIR***-***8900 *Shimadzu *spectrophotometer *with KBr discs; Ultraviolet (UV) spectra were recorded on Hitachi U-3200 spectrophotometer; EI-MS spectra were measured in an electron impact mode on Varian MAT 112 or MAT 312 spectrometers. Chromatographic materials were silica gel (25-40 μm; LiChroprep® Si 60) and Sephadex LH-20 (Pharmacia, Inc., Piscataway, NJ, USA). TLC detection was achieved by spraying the silica gel plates with ferric chloride and cerium sulphate in 10% aq.H_2_SO_4_, followed by heating.

*Plant material*

Aerial flowering parts of *Achillea tenuifolia *Lam. (Asteraceae) was collected from Zanjan province (Iran). Plant material was identified by M.Kamalinezhad, plant taxonomist and a voucher specimen deposited in the herbarium of the Faculty of Pharmacy, and Pharmaceutical Sciences in Shahid Beheshti University of Medical Sciences (Iran). 

*Extraction and isolation*

The powder of the air-dried plant material (4 kg) was soaked in methanol (15 L × 3) at room temperature for 3 days, (By three times), and the resulting extract was concentrated to a gum (300 g). Methanolic extract was dissolved in distilled water and defatted with petroleum ether. The defatted aqueous extract was further fractionated with dichloromethane and n-butanol. The dichloromethane fraction (50 g) was chromatographed on normal column using gradient mixtures of Hexane: EtOAc (0 → 100%) followed by methanol to afford ten fractions: Fr.1-Fr.10. Fr. 1 contained oils and fatty acids (inferred from ^1^H-NMR spectra). Fr. 2 eluted with Hexane/EtOAc (95: 5) was purified by two times preparative TLC with the eluent system of Hexane/ Me_2_CO (8: 2) to give compound 1. Rich chlorophyl fracrions (Fr. 3-5), were put aside and Fr. 6 eluted with Hex / EtOAc (8: 2) was rechromatographed on silica gel (Hexane/ Me_2_CO, 6: 4) to render several fractions: Fr. 6a-h. Then, Fr. 6a, contained mixtures of flavonoids and pigments, was further separated on PLC to yield Fr. 6a8 as compound 2. Finally, fraction eluted with EtOAc / MeOH (9: 1) was loaded on normal columns using Me_2_CO / MeOH (9: 1) to obtain pure compound 3. 

*β*-sitosterol (1). White crystals, m.p. 138-142˚ C, ^1^H-NMR (400 MHz, CDCl_3_): 0.66 (3H, s, H-18), 0.79 (3H, br d, H-26), 0.81 (3H, d, J=7.6 Hz, H-27), 0.83 (3H, bd, H-24), 0.90 (3H, d, J= 6.4 Hz, H-21), 0.99 (3H, s, H-19), 3.50 (1H, m, H-3), 5.33 (1H, m, H-6). 13C-NMR (100 MHz, CDCl_3_): 140.8(C-5), 121.7 (C-6), 71.8 (C-3), 56.8 (C-17), 56.1 (C-14), 50.2 (C-9), 45.9 (C-24), 42.3 (C-13, C-4), 39.8 (C-12), 37.3 (C-1), 36.5 (C-10), 36.1 (C-20), 34.0 (C-22), 31.9 (C-8, C-7), 31.7 (C-2), 29.2 (C-25), 28.2 (C-16), 26.1 (C-23), 24.3 (C-15), 23.1 (C-28), 21.1 (C-11), 19.8 (C-26), 19.4 (C-19), 19.1 (C-27), 18.8 (C-21). EIMS *m/z *(rel. int.): 414 [M]^+^ (22), 329 [M-C_5_H_7_-H_2_O]^+^ (7), 303 [M-C_7_H_9_-H_2_O]^+^ (9), 273 (5), 255 (7), 231(7), 213 (11), 161 (16), 119 (25), 107 (44), 105 (43), 95(49), 79(30), 71(36), 69(63), 67(40), 57 (99), 55 (100).

5-Hydroxy, 4’, 6, 7–trimethoxyflavone (2). Pale yellow powder. m.p.: 185°C, UV max (MeOH): 276, 328 nm; ^1^H-NMR (500 MHz, CD_3_OD) δ 3.83,3.89,3.98 (each 3H, S, OMe), 6.70 (1H, S, H-8) , 6.83 (1H, S, H-3 ), 7.08 (2H, d, J=9 HZ , H-3’ ,5’ ), 7.97 (2H ,d, *J*=9 HZ , H-2’,6’). ^13^C-NMR (125 MHz, CD_3_OD) δ 55.5 (4’-OMe),56.2 (7- OMe), 60.8 (6-OMe), 90.5(C-8),104 (C-3), 106.4 (C-10 ), 114.4 (C-3’,5’), 123.4 (C-1’ ), 127.9 (C-2’,6’ ), 132.5 (C-6 ), 153.0 (C-5 ), 153.2 (C-9 ) ,158.7 (C-7) ,162.6 (C-4’ ), 163.9 (C-2 ), 182.6 (C-4 ). HREI-MS *m/z *328.0931 (calcd. for C_18_H_16_O_6_, 328.0946); EI-MS *m/z *(%): 328 (100), 313 (99), 285 (25), 282 (22), 181 (26), 153 (73), 135 (21), 133 (37), 85 (20), 83 (44), 71 (29), 69 (90), 57 (49), 55 (36). 

Methyl-galate (3). White crystals, Mp: 197-199 °C. IR (KBr) *γ *max: 3500, 3300, 1690, 1610, 1530, 1460, 1435, 1310, 1250, 1190, 1040 cm^-1^. ^1^H-NMR (400 MHz, CD_3_OD): 3.80 (3H, s), 7.04 (2H, s). HREI-MS *m/z*: 184.0362 (calc. for C_8_H_8_O_5_, 184.0372, Δ -5.2 ppm), EIMS *m/z *(rel. int.): 184 [M]^+^ (81), 154 [M-MeOH]^+^ (10), 153 [M-MeO]^+^ (100), 125 [M-COOMe]^+^ (23), 107 (5), 79 (8), 44 (9).

*Determination of stoichiometry of metal-ligand complexes*

The mole-ratio method has allowed us to determine the composition of the complexes in solution from spectrophotometric spectra. In this method, the flavonoid stock solution (1.0 × 10^-3^ M) was prepared successively in methanol. Iron(III), Cupper(II) and Zinc (II) solutions as metal cation solution were prepared in concentration of 1.0 × 10^-3^ M from reagent grade Fe (NO_3_)_3_, CuCl_2_ and ZnSO_4_ from Merck company. A concentration of 1.0 × 10^-4^ M of flavonoid was diluted from stock solution and kept constant. Typical titration experiments were performed by sequential additions of 33 μL of metal ion solution (1.0 × 10^-3^ M) to the same 3 mL flavonoid solution (1.0 × 10^-4^ M) in a quartz cuvette. UV/Vis spectra were recorded on a Perkin Elmer Lambda 25 spectrometer at 25 °C and absorbance of each solution was then measured at absorbance maxima and plotted vs. metal ion/ ligand (M/L) molar ratios. The break point in the plot is corresponded to the mole-ratio of the metal ion in the ligand-metal complex (10, 11).

*Computational molecular modeling*

All calculations were run on a TOSHIBA laptop computer with Genuine Intel(R) CPU running Windows XP operating system. The Chem-Draw Ultra version 9.0 (Chem-Office 2005, Cambridge-Soft Corporation; Cambridge, MA) software was employed for drawing the initial 2D complexes structures. The generated 2D structures were transferred to MOE and Gaussian 98 software for further geometry optimization. Applying PM3 semi-empirical calculations, the 3D geometry optimization process was run many times with different starting points and finally the theoretical heat of formation of each complex were calculated separately (10).

## Results and Discussion

Compound 2 was isolated from dichloromethane soluble part of the methanolic extract of *Achillea tenuifolia*. The HREI-MS exhibited the molecular ion peak at 328.0931 [M]^+^ , corresponding to the molecular formula C_18_H_16_O_6_ consistent with eleven degrees of unsaturation. The UV spectrum showed absorption maxima at 276 and 330 nm, characteristic of flavone derivatives (12). The bathochromic shifts of Band I (in MeOH) to Band Ia (in AlCl3/HCl) was 30 nm in agreement with those recorded for 5-hydroxy flavones (35-55 nm) and in contrast with 3-hydroxy-flavones (and 5-hydroxy-3-hydroxy-substituted flavonols), which is around 50-60 nm (12). EIMS spectrum showed *m/z *328 as a base ion together with fragment ions of *m/z *181 and 133, characteristic retro-Diels-Alder (RDA) fragments for a flavone-skeleton with two methoxyl and one hydroxyl groups on ring A and one methoxyl group on ring B (13, 14). ^13^C NMR showed 18 carbon signals including three methoxyl carbons δ 55.5 (4’-OMe), 56.2 (7- OMe), 60.8 (6-OMe), 14 olefinic carbones, and a carbonyl (182.6 ppm) carbon. The ^1^H-NMR showed two doublets, each of two proton integration at δ 7.08 and 7.97 ppm with coupling constant 9 Hz. These were assigned to H-3’, 5’ and H-2*'*, 6’ respectively, which were ortho to each other in ring B. The same spectrum showed two singlets, each integrated for one proton at δ_H_ 6.70 (H-8) and 6.83 (H-3), and one proton at δ_H _8.54 ppm, which could be assigned as a hydroxyl group on C-5 position of flavone structure. Therefore, based on similar NMR data, published before, compound 2 was deduced to have a structure as 5-hydroxy, 4*'*,6,7 -trimethoxy flavone (14, 15). 

In the same manner, comparison of the NMR and Mass spectral data of compound 1 and 3 with those published before in literature allowed us to establish the structures of these two other known compounds as *β*-sitosterol (1) and methyl-galate (3) which was confirmed by co-TLC with their authentic samples (4,15-18).

*Chemotaxonomic significance*

The isolation of 5-hydroxy, 4*'*,6,7 – trimethoxy flavone (salvigenin) was in agreement of a survey of detailed favonoid distribution within Achillea species which revealed a strong tendency towards formation of (polymethoxy) favonoids and specially 6-methoxylation as a basic chemical trend within the genus Achillea. It was also in accordance with the reported examination of exudate flavonoids have already been found in *A. ptarmica, A. millefolium , A. nobilis, A. ageratum, A. ochroleuca, A. babounya, A. clavennae *and *A. talagonica *which would merit taxonomic recognition (4, 18-19). 

*Detemination of stoichiometry of metal-ligand complexes*

**Figure 2:**
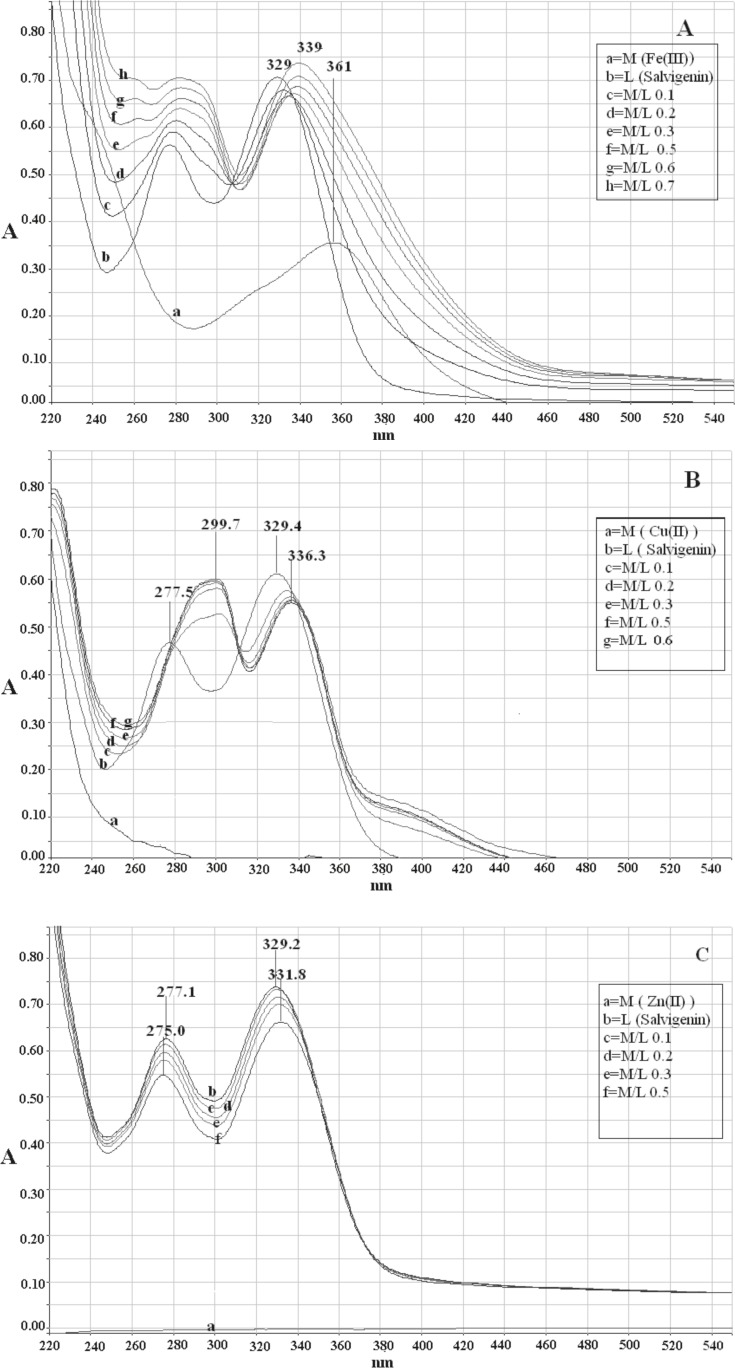
Electronic absorption spectra of (a) salvigenin (1.0 × 10^-4^ M) in methanolic solution in absence and presence of Fe(III) without pH control, (b) salvigenin (.0 × 10^-4^ M) in methanolic solution in absence and presence of Cu(II) without pH control, (c) salvigenin (.0 × 10^-4^ M) in methanolic solution in absence and presence of Zn(II) without pH control

Two major absorption bands with maxima at 329 nm (band I) and 277 nm (band II) characterized the UV-Vis spectrum of salvigenin in methanolic solution without pH control (Figure 2(a)). Before studying the interaction of metal ions with compound 2 (salvigenin), the control studies were done on quercetin and the results were compared to literature to ensure that the obtained results are true. The addition of Fe(III) to quercetin (Sigma-Aldrich Corp., St. Louis, MO, USA), as positive control resulted in significant change of the absorbance spectrum with the appearance of a new band centered on 428 nm with bathochromic shift of about 58 nm from the original band in the absence of Fe(III). Similarly, the absorbance of band I of salvigenin decreased at 329 nm and a new band, which increased with the amount of added Fe^+3^ ions, appeared at 339 nm. The higher Fe (III) concentration, resulted in higher amount of the salvigenin and quercetin complexes in solution and the intensity of the absorption band at the long wavelength was increased. The stoichiometry ratio of Fe(III) with salvigenin and quercetin without pH control were 1:2 and 1:1, respectively. In the same manner complexation of Cu (II) with flavonoids produced a bathochromic shift in band II of 18 nm and 22 nm together with red shift at the position of band I of 54 nm and 7 nm for quercetin and salvigenin (Figure 2 b), subsequently with stoichiometry ratio of 1:2. Under similar conditions, in the Zn(II) complexation of quercetin, the experimental results showed 12 nm and 52 nm red shift in band II and band I with mole-ratio of 1:1, while surprisingly, no changes were observed in the positions of both the bands in the 5-hydroxy-6,7,3′-trimethoxyflavone spectrum even in higher concentrations (Figure 2c).

*Computational molecular modeling*

**Figure 3:**
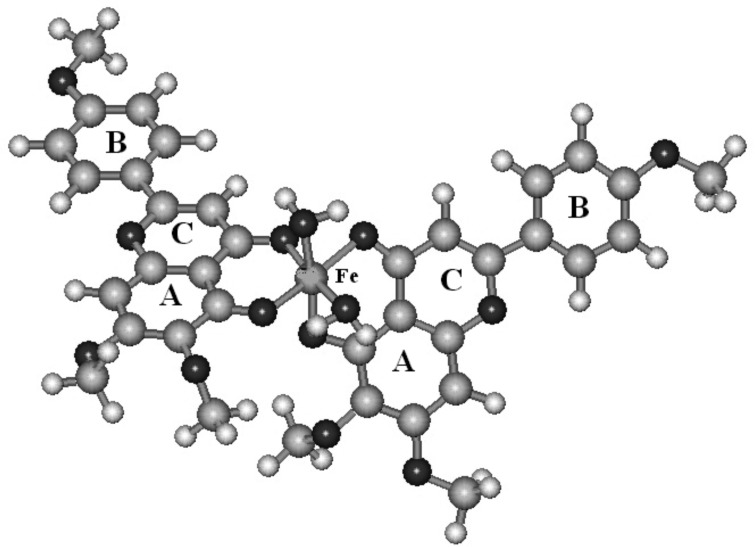
The proposed structure of Fe (III) complexation with salvigenin

The derivation of theoretical heat of formation proceeds from the optimized chemical structure of the investigated complexes (Figure 3). The calculated heat of formation for each studied complexes are summarized in table 1. The water molecules have been added to achieve octahedral structures for complexes which have two bounded ligands. It can be seen that calculated heat of formation have negative values for the studied complexes. 

**Table 1:** Heat of formation of proposed salvigenin-complexes by using semi-empirical PM3 calculations

**Complex **	**Heat of formation **
Fe (lig)_2_(H_2_)_2_	689.732-
Cu (lig)_2_(H_2_)_2_	573.543-

## Conclusion

We investigated the complexation of salvigenin with Fe(III), Cu(II) Zn(II), the most abundant type of metal ions in the body. Applying molar-ratio method, it was determined that stoichiometric ratio of salvigenin with Fe(III) and Cu(II) were as Fe (Salvigenin)_2_ (H2O)_2_ and Cu (Salvigenin)_2_ (H2O)_2_ in methanolic solution without pH control, while zinc ions didn`t show significant complexes with salvigenin. These results were confirmed more, by computational molecular modeling of the structure of proposed ligand-complexes by semi-imperical PM3 calculations, which determined negative heat of formation for the complexes Fe(III) and Cu(II) ions as -689.7 and -573.5, showing the affinity of salvigeninthe in the following order: Fe(III)> Cu(II) >> Zn(II). 

In comparision with quercetin, the Fe-binding properties with more affinity than Cu(II) ions without chelating Zn(II) proposed salvigenine as an iron-chlating candidate in dietary supplementary medicine especially for patients with high iron levels like thalassemia, and wilson`s disease with toxic levels of copper in the body. 
